# Exercise as a welfare strategy? Insights from horse (*Equus caballus*) owners in the UK

**DOI:** 10.1017/awf.2025.11

**Published:** 2025-03-04

**Authors:** Cynthia Joanne Naydani, Tamsin Coombs

**Affiliations:** 1 Royal (Dick) School of Veterinary Studies, University of Edinburgh, Edinburgh EH25 9RG, UK; 2 Department of Animal and Veterinary Sciences, Scotland’s Rural College (SRUC), Edinburgh EH9 3JG, UK

**Keywords:** animal welfare, behaviour change, COM-B, equine, management, obesity

## Abstract

Obesity and its comorbidities (e.g. laminitis) are identified as major welfare issues among domestic equids in the United Kingdom (UK) and abroad. Weight-management typically focuses on restricting consumption (e.g. limiting pasture grazing), often facilitated through stabling. This leads to social isolation, prompting other welfare issues. Increased exercise may be a preferable solution for equine obesity, if viable. The aims of this study were to explore horse (*Equus caballus*) owner perceptions regarding the importance of exercise, and to investigate how exercise provision related to welfare outcomes. Data obtained via an online survey from 804 UK horse owners indicated that most respondents’ horses were managed in obesogenic conditions, and 40% were owner-reported as overweight/obese. Exercise-related variables (e.g. if a horse was ridden) correlated with physical health problems, including decreased reports of laminitis and Equine Metabolic Syndrome. Approximately 90% of respondents reported that barriers outside of their control substantially limited opportunities to provide human-led exercise (e.g. riding, lunging). Analysis of a hypothetical weight-management scenario found owners with horses at livery yards felt significantly less able to increase horses’ self-directed exercise (e.g. free movement in fields/pastures) than owners keeping horses on their own properties. These findings indicate that while increased exercise may improve welfare, owner knowledge is not the only barrier that must be overcome to implement this prospective solution. Both human-led and self-directed exercise appear limited by a lack of opportunities available to horses and humans. These results justify future investigations into alternative management strategies as potentially viable methods of increasing exercise to improve welfare.

## Introduction

Obesity is one of the most pressing welfare issues currently facing domestic horses (*Equus caballus*) in the United Kingdom (UK) (Rioja-Lang *et al.* 2020). It is associated with conventional management strategies that centre around individual stabling and limited turnout (Hockenhull & Creighton 2014). ‘Turnout’ refers to access to outdoor areas such as pastures, fields, or paddocks, which provide opportunities for natural behaviours, including free movement, socialisation, and grazing. These 3Fs: freedom, friends, and forage, are fundamentally important for optimising quality of life in horses (Hall & Kay 2024). Provision of adequate turnout can contribute directly and positively to each component of Mellor *et al.*’s (2020) Five Domains model, which explains that an animal’s welfare state will be impacted by his or her physical environment, behavioural interactions, nutrition health, and mental state. However, while grazing is a natural behaviour for horses and important for the health, nutrition, and mental state domains, typical grass-based turnout environments may be obesogenic, as they can facilitate overconsumption of nutrient-rich forage (Longland & Byrd 2006). This can therefore be detrimental to the health domain of welfare. Obesity risk can be further exacerbated by owner over-estimation of workload, leading to excess provision of concentrated feeds (Hale *et al.* 2016). Owners may also provide unnecessary concentrated feeds to fulfil a perceived psychological desire in their horses, rather than a physical need (Karasu & Rogers 2024). While obesity is a standalone health problem, it may also lead to debilitating comorbidities including laminitis and Equine Metabolic Syndrome (EMS) (Geor 2010).

Recommended weight control strategies commonly focus on nutritional management, including restricted grazing via individual stabling, which can increase social isolation and limit movement (Gill *et al.* 2017; Ruet *et al.* 2019). This focus on confinement and reducing dietary intake contrasts with the evolutionary history of horses, whereby animals travelled in groups over large home ranges, grazing continuously on diverse low-nutrient forages (McGreevy 2012). Despite domestication, horses remain physiologically adapted and behaviourally motivated to pursue wild-type activity budgets (McGreevy 2012). Restrictive housing and dietary management can lead to gastrointestinal issues including ulcers (Aranzalez & Alves 2013) and colic (Mills & Clarke 2007), as well as the development of unwanted behaviours such as stereotypies (Henderson 2007).

There is therefore a need to approach the challenge of weight management from a holistic perspective, facilitating species-normal behaviours while contributing positively towards physical health. A potential solution lies in promoting more movement, focusing on increasing energy expenditure rather than solely on decreasing energy intake. Increased activity levels in horses can be achieved through structured human-led exercise (HLE), encompassing ridden work, lunging, and in-hand groundwork. Alternatively, access to appropriate turnout can enable horses to undertake self-directed exercise (SDE) through free movement, potentially resulting in fitness benefits that equal or outweigh those experienced by horses who are stabled and only receive HLE (Graham-Thiers & Bowen 2013). To mitigate the risk of obesity, as outlined above, however, turnout environments should ideally limit intake of high-sugar pasture grasses, while still allowing the near-constant grazing that exemplifies normal equine consumption patterns. Several alternative grazing systems are now being applied in the UK, including track systems, whereby horses follow paths to access resources including hay, water, and shelter, which are distributed throughout the habitat (Jackson 2006; Furtado *et al.* 2021). These systems can encourage movement and facilitate socialisation, while managing dietary intake, contributing positively to overall equine well-being (Kirton *et al.* 2024). Another alternative is strip-grazing, which involves cordoning off sections of pasture, gradually allowing access to ungrazed areas, thus controlling consumption (Cameron *et al.* 2022). However, strip-grazed ponies preferentially graze fresh grass (Cameron *et al.* 2022), and move less than those on track systems (Kirton *et al.* 2024), indicating that track systems may be preferable for welfare.

Understanding horse owner perceptions is an important step in assessing the potential for HLE and/or SDE to contribute positively to equine welfare. Lack of owner knowledge is often cited as the root cause of equine mismanagement, and subsequent welfare issues (Horseman *et al.* 2016; Rioja-Lang *et al.* 2020). However, the COM-B model of behaviour change states that behaviours are shaped through a combination of capability, motivation and opportunity (Michie *et al.* 2014). The COM-B model therefore provides a holistic lens through which to better understand equine management. Importantly, the COM-B framework explains that opportunity is necessary for a behaviour to manifest, such that, even if capability and motivation promote a behaviour, a lack of opportunity will prevent it (Michie *et al.* 2014). This is highly relevant when investigating horse owner perceptions regarding exercise as a means to improve welfare, as factors outside the owner’s control may restrict opportunities for both SDE and HLE.

The aim of this project was to gain insight into UK horse owners’ perceptions regarding exercise as an opportunity to improve the welfare of their horses. Specific objectives were to: (i) describe prevailing current practices regarding both HLE and SDE; (ii) investigate correlations between exercise-related variables and prevalence of owner-reports of physical and behavioural issues; (iii) determine if horse owners want to provide more exercise than their horses currently undertake, and if so, what barriers prevent this?

## Materials and methods

### Study design

This study received ethical approval from the University of Edinburgh’s Human Ethics Review Committee (HERC_697_21). Data were collected via a digital questionnaire (Jisc Online Surveys) which was distributed via online social media platforms, receiving 804 responses over four weeks. The survey was open to adults residing in the UK who had owned, loaned and/or shared a horse over the past 12 months. The survey specified that ‘horse’ referred to either a horse (> 14.2 hh) or pony (≤ 14.2 hh). The same language is used here. Participants provided informed consent, confirming that they understood the purpose of the research and what their data would be used for. They were informed of their right to withdraw during survey completion though, due to the anonymous nature of the survey, responses could not be revoked following submission.

The survey consisted of 34 closed-answer questions (Supplementary material). Respondents disclosed basic demographic information and information regarding their equine experience, before answering questions about (one of) their horse(s). Respondents with multiple horses were instructed to respond considering the animal whose stable name came first alphabetically. Questions covered equine demographics, use, and ownership status. Respondents self-reported their horse’s physiological and psychological health over the previous 12 months, including perceived presence of over/underweight, physical health issues, and observations of behavioural welfare indicators (presence of locomotor stereotypies and/ or aggression towards humans).

Management questions included where the horse was kept, including type of livery if applicable, and factors influencing facility choice. Information regarding opportunities for SDE was gathered via questions on turnout type, size, access (hours per day), number of companions, forage availability (hours per day) and type(s) of enrichment items. Questions were repeated for ‘summer’ and ‘winter’ conditions, although these seasons were not specifically defined to allow for individualisation (e.g. some livery yards use winter turnouts from 1 October to 1 May, whereas others base seasonal turnout on prevailing weather conditions). Respondents specified their level of control over turnout conditions and relayed their satisfaction with this control. Data were gathered regarding HLE, such as being ridden or lunged, undertaken by respondents’ horses. Respondents identified the type(s) of HLE their horses undertook in summer and winter, and the frequency, duration, and intensity of exercise. They reported their levels of satisfaction with their horses’ workloads and identified major barriers to providing additional HLE.

To analyse beliefs, a hypothetical ‘real-world’ scenario was then described whereby a fictional horse was at risk of developing health issues due to excess body condition (Supplementary materials; Q34). A veterinarian recommended three potential solutions: (i) increasing HLE; (ii) daytime stabling plus use of a grazing muzzle during turnout (thus reducing but not eliminating SDE); or (iii) modifying the turnout area to promote SDE, maintain socialisation ability, and limit access to high-sugar grass. Respondents identified the solution they thought was: (i) most effective; (ii) most realistic in their current situations; (iii) preferable from their horse’s perspective; and (iv) ideal if all were equally possible. The design of this question was guided by the COM-B model of behaviour change (see previously) (Michie *et al.* 2014).

### Data preparation

Data were exported to Microsoft Excel® and coded for analysis. Specific breeds were grouped into three breed types: hot-bloods (thoroughbred, Arab, Anglo-Arab), sports horses (e.g. Warmblood, Irish sports horse, thoroughbred cross, gaited breeds) and natives (e.g. Highland, cob, Shetland, draught breeds). HLE measures were coded as detailed below. Box walking, fence walking, and weaving were combined as ‘locomotor stereotypies’.

#### Human-led exercise measures

Responses for summer and winter HLE frequency, duration, and intensity were coded, based on workload guidance by Frape (1998; p 177) and Clayton (1991; pp 80–81,111–113, 159) (Table 1). A relative measure of total HLE was calculated by summing each exercise component. For example, a horse ridden twice per week (3) for an average of 40 min (2) at low intensity (1) would receive a score of 3 + 2 + 1 = 6. A horse ridden five days per week (4) for over an hour (3) at a moderate intensity (2) would receive a score of 4 + 3 + 2 = 9.

**Table 1.** Coding of variables measuring human-led exercise in horses, consisting of assigned values for categories of owner-reported (n = 802) exercise frequency, duration, and intensity

Assigned value	Variable		
	Exercise frequency	Exercise duration	Exercise intensity
0	Not in work	Not in work	Not in work
1	Once a month	Under half an hour	Low (Mainly walking, some trot)
2	Once a fortnight	30–60 min	Moderate (Mainly trot, some canter)
3	1–2 days per week	Over an hour	High (Lots of canter, some jumping)
4	3–5 days per week	-	Very high (Mainly fast canter, galloping and/or jumping)
5	6–7 days per week	-	-

#### Environmental enrichment

Respondents selected from a list all the environmental enrichment features present in their horse’s turnouts (Supplementary material; Q23g, Q24g). The total number of selected items was used as a proxy measure of environmental complexity.

#### Welfare indicators

Binary scores (1 = present, 0 = absent) were used to denote owner-reported occurrences of negative welfare indicators. Physiological indicators were: (i) if the owner considered their horse’s average body condition over the past 12 months to be ‘overweight’, and if, within the past 12 months, the owner reported their horse had been diagnosed/treated/managed for (ii) laminitis; or (iii) EMS. Behavioural indicators were (i) owner-reported presence of locomotor stereotypies (encompassing box walking, fence walking, and weaving), and (ii) observed displays of aggression towards humans, again within the past 12 months.

### Data analysis

Statistical analyses were conducted in Minitab® 19 using an alpha value of 0.05. Stepwise binomial logistic regression (BLR) was used to determine relationships between independent variables and welfare indicators, after Hosmer-Lesmeshow goodness-of-fit tests confirmed regression assumptions were met. BLR was also used to identify variables affecting respondent satisfaction with their control over their horse’s opportunities for SDE via turnout conditions, and their satisfaction with provided amounts of HLE. Factors included in BLR models were chosen based on previous literature, and predictions regarding impacts of movement-related variables on welfare outcomes. Multicollinearity was assessed by ensuring the condition number for the overall model was less than 100, as values over 100 indicate moderate multicollinearity (Montgomery *et al.* 2012). Highly correlated predictors were removed from any models with moderate multicollinearity to remove redundancy from the model. For all BLR analyses, continuous variables with Odds Ratios (OR) > 1 denotes the given condition is more likely to occur as the predictor increases, and < 1 indicates the condition is less likely to occur as the predictor increases. Chi-squared tests investigated responses to the hypothetical scenario question. These tests considered yard type (livery versus own property) to be the predictor variable, and the frequency of each respondent-selected intervention (muzzling, increased HLE, altered field setups, or ‘unsure’) to be the dependent variable. A separate Chi-squared test was conducted for each component of the scenario: which intervention respondents believed would be: (i) most effective; (ii) most realistic; (iii) preferable from their horse’s perspective; and (iv) ideal if all were equally possible.

## Results

### Respondent demographics

Of the 804 respondents, the majority resided in England (n = 430; 53.5%) or Scotland (n = 333; 41.4%), were female (n = 784; 98%), 35–59 years of age (n = 478; 59.6%), and educated to at least degree level (n = 478; 59.6%). Most respondents self-identified as leisure owners (n = 670; 83.3%). Most respondents were experienced, with over a decade of involvement with horses (n = 718; 89.3%). Just 4.5% (n = 36) of respondents were caring for their first and only horse at the time of survey completion, while most (n = 505; 62.8%) were caring for multiple horses at the time of completion.

### Horse information

Most respondents answered for a horse, not a pony (n = 591; 73.4%). Natives were the most populous breed type (n = 349; 43.4%), followed by sports horses (n = 334; 41.5%) then hot-bloods (n = 121; 15.0%). Most horses were ridden (n = 645; 80.2%), and owned (n = 756; 94%), rather than shared or loaned. The word ‘owner’ is therefore used henceforth in reference to any respondent.

**Figure 1.**
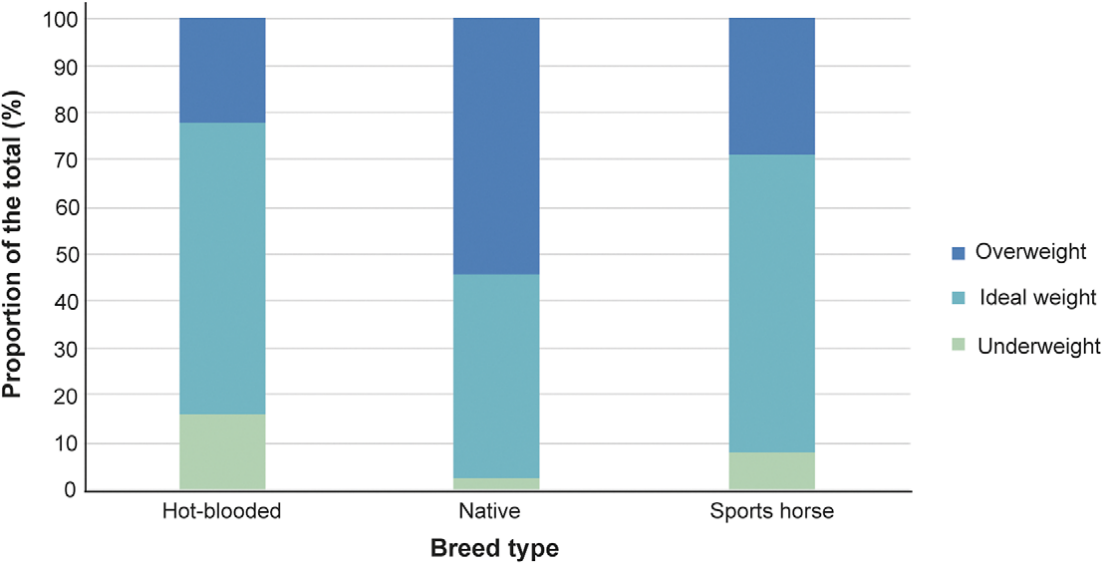
Average horse body condition as reported by each horse’s owner (n = 802) over the previous 12 months, as a function of horse breed type.

Most respondents reported that their horses gained weight easily (n = 492; 61.2%). Despite 314 horses (39.1%) having been reported as overweight or obese throughout the past year, only 101 (12.6%) had been diagnosed, treated, or managed for excess weight within the same time-frame. Owner-reported laminitis affected 54 horses (6.7%), while fewer horses were reported to have EMS (n = 27; 3.4%). Natives were more commonly overweight than hot-bloods or sports horses, and within natives, more were overweight than not (Figure 1). Locomotor stereotypies were reported in 79 horses (9.8%) and 108 horses (13.4%) had displayed aggression towards humans.

Most horses were either kept at livery yards (n = 380; 47.3%) or at the respondent’s own property (n = 354; 44%), while the remaining 70 horses (8.7%) were kept elsewhere. Among horses on livery yards, 248 (65.3% of livery horses, 30.8% of total horses) were on ‘Do-it-yourself’ (DIY) livery, 72 (18.9%/9%) were on part-livery and 60 (15.8%/7.5%) were on full livery (see Supplementary material; Q20a for livery descriptions). Most horses had lived at their current yard for over a year (n = 629; 78.2%). The two most common reasons for changing facilities were location (n = 196; 24.4%) and to improve the horse’s living conditions (n = 188; 23.4%). Only 43 respondents (5.3%) changed yards primarily to improve riding facilities.

### Self-directed exercise

**Figure 2.**
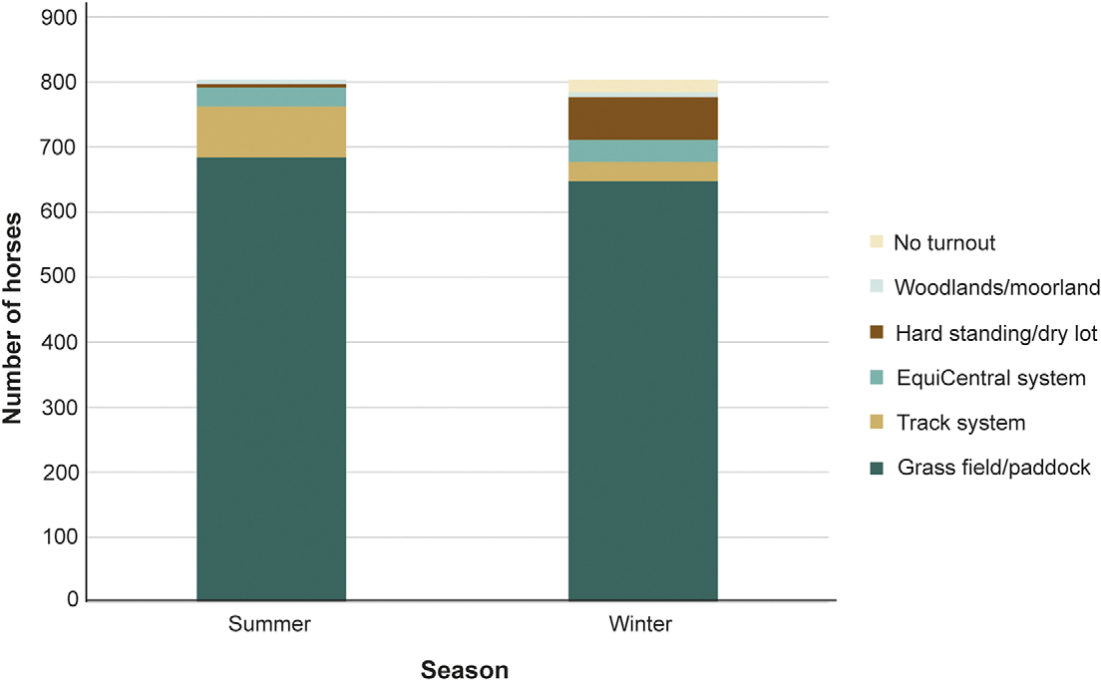
Turnout type for survey respondents’ (n = 802) horses in summer and winter seasons.

Most horses were turned out into grass fields year-round (Figure 2).

**Figure 3.**
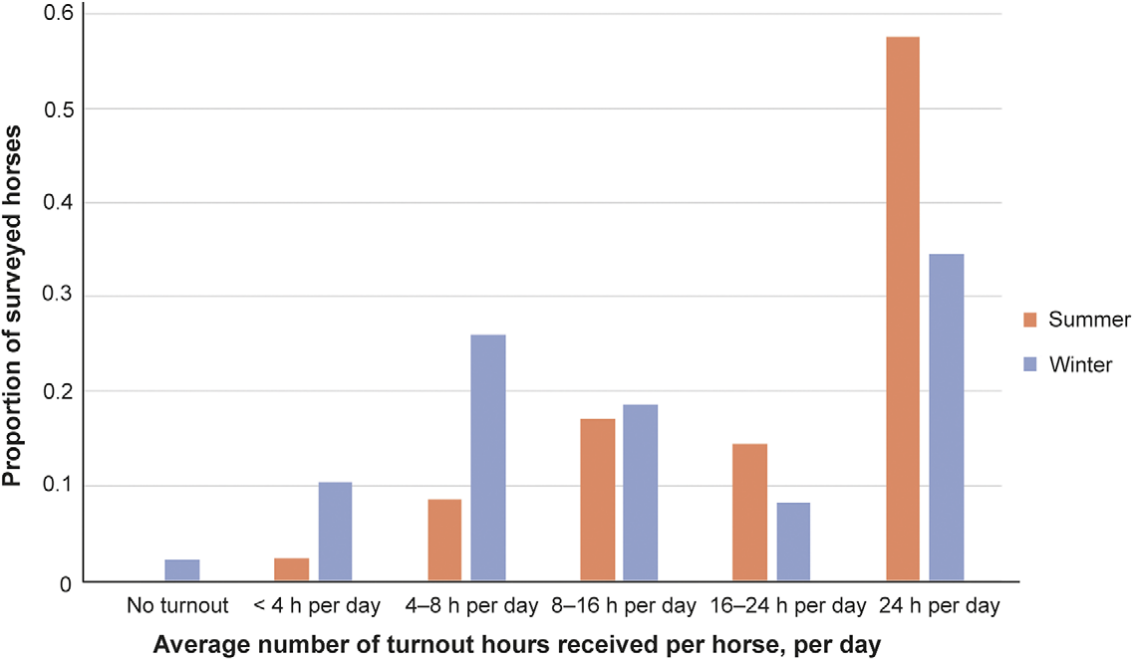
Average seasonal turnout duration for horses as reported by their owners (n = 802).

Over summer, over half of the horses (n = 462; 57.5%) were turned out 24 h per day, and all received a degree of turnout (Figure 3). Over winter, 278 horses (35.4%) had access to turnout 24 h per day, and 18 (2.2%) received no turnout (Figure 3). Environmental complexity was similar between summer and winter turnouts. The most common number of enrichment item types was two or three, and very few turnouts had eight or more (range 0–10).

Owners who did not keep their horses on livery were more likely to be satisfied with their level of control over turnout conditions than those whose horses were on livery (OR = 9.35; *P* < 0.001), and respondents were more likely to be satisfied with increased: hours of turnout (OR = 1.26; *P* = 0.002), environmental complexity (OR = 1.51; *P* < 0.001), winter forage availability (OR = 1.29; *P* = 0.027), and decreased: total number of behavioural issues (OR = 0.80; *P* = 0.009), and number of conspecifics in winter turnouts (OR = 0.78; *P* = 0.015).

### Human-led exercise

Year-round, the most popular types of HLE were hacking (summer: n = 570; 70.9%; winter: n = 530; 65.9%), flatwork (n = 511; 63.6%; n = 478; 59.5%) and polework (n = 426; 53%; n = 366; 45.5%) (Table 2). In summer, 78 horses (9.7%) were not in work, while in winter 109 horses (13.6%) did not receive HLE.

**Table 2.** Human-led exercise types for horses in summer and winter, as reported by their owners (n = 802). Respondents could select multiple responses, as applicable

	Summer		Winter	
Activity	Number of respondents	Percent of respondents (%)	Number of respondents	Percent of respondents (%)
Flatwork/dressage	511	63.6	478	59.5
Showjumping/arena jumping	196	36.8	233	29
Cross country jumping	204	25.4	40	5
Polework	426	53	366	45.5
Conditioning work (e.g. gallop sets, intervals, hillwork)	248	30.8	98	12.2
Hunting/team chasing	11	1.4	31	3.9
Showing: ridden	66	8.2	15	1.9
Showing: in-hand	51	6.3	10	1.2
Driving	13	1.6	11	1.4
Hacking	570	70.9	530	65.9
Endurance riding	70	8.7	17	2.1
Multi-day trekking	20	2.5	5	.6
Lunging/long-lining	384	47.8	325	40.4
Other in-hand exercise	346	43	302	37.6
Other exercise	48	6	40	5
None of the above – horse is not in work	78	9.7	109	13.6

**Figure 4.**
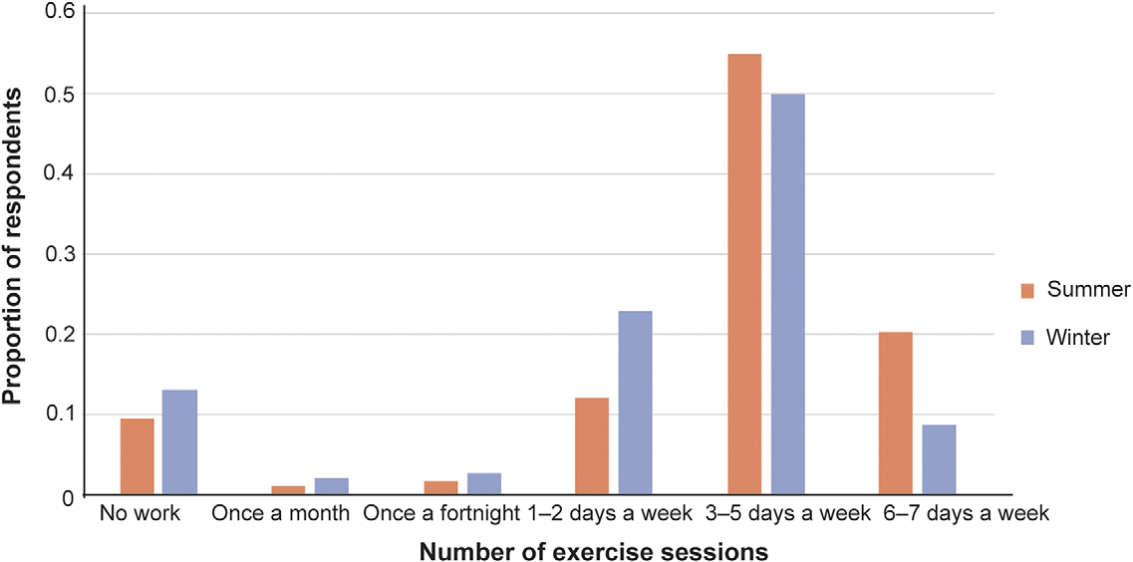
Frequency of human-led exercise performed by horses in summer and winter, as reported by their owners (n = 802).

**Figure 5.**
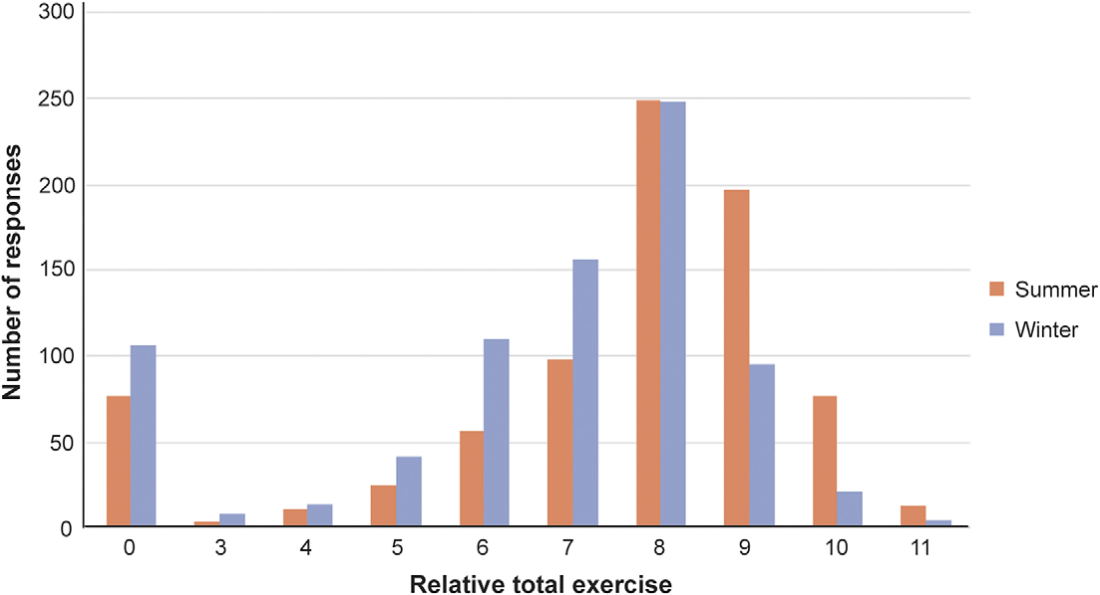
Total amount of human-led exercise received by horses by season, according to their owners (n = 802). Relative exercise levels were quantified as the sum of coded data for frequency, duration, and intensity of exercise. For example, a horse exercised twice per week (3) for an average of 40 min (2) at low intensity (1) would receive a score of 3 + 2 + 1 = 6.

Most horses received HLE three to five times per week in both summer and winter (Figure 4). Total HLE levels were similar between the two seasons, though slightly lower in winter (Figure 5).

When asked to identify the main barriers limiting HLE, only 83 respondents (10.3%) reported there were none. The top three barriers were weather (n = 550; 68.4%), time (n = 434; 54%) and suitable facilities (n = 284; 35.3%) (Table 3).

**Table 3.** Barriers to the provision of human-led exercise in horses, according to their owners (n = 802). Respondents were able to select up to three responses, as appropriate

Barrier	Number of respondents	Percent of respondents (%)
Access to facilities/suitable areas to exercise	284	35.3
Access to other people to ride with (including instructor)	100	12.4
Time	434	54
Weather	550	68.4
Horse age	132	16.4
Horse health/soundness	159	19.8
Horse behaviour	65	8.1
Rider/handler health or fitness	93	11.6
Rider/handler confidence or skill level	53	6.6
General human desire/motivation to ride or exercise the horse	171	21.3
Other barrier	38	4.7
None of the above – can exercise as much as desired	83	10.3

Respondent satisfaction with the amount of HLE received by horses was only significantly predicted by yard type, irrespective of season (Table 4). Respondents whose horses were on livery were more likely to be satisfied than those with horses on their own property (summer: OR = 0.69; *P* = 0.033; winter: OR = 0.68; *P* = 0.017) or kept elsewhere (summer: OR = 0.52; *P* = 0.022; winter: OR = 0.53; *P* = 0.016).

**Table 4.** Significant predictors of horse owner (n = 802) satisfaction with the amount of human-led exercise received by their horses

Satisfied = 1; Desire more control = 0						
Independent variable	Level	Co-efficient	SE co-efficient	*P*-value	Odds ratio	95% CI
Yard type (summer)	Livery	Baseline				
	Own	–0.372	0.174	**0.033**	0.689	0.49, 0.97
	Other	–0.646	0.282	**0.022**	0.524	0.76, 2.26
Yard type (winter)	Livery	Baseline				
	Own	–0.387	0.162	**0.017**	0.679	0.49, 0.93
	Other	–0.644	0.267	**0.016**	0.525	0.31, 0.89

### Welfare indicators

The only significant predictor of a horse being owner-identified as overweight over the past year was breed type: hot-bloods were less likely to be reported as overweight than natives (OR = 0.23, 95% CI [0.14, 0.39]; *P* < 0.001] as were sports horses (OR = 0.33, 95% CI [0.23, 0.48]; *P* < 0.001) regardless of turnout type or ridden status.

Ridden horses were less likely to be reported to be laminitic than unridden horses (OR = 0.38; *P* = 0.002), and laminitis was less likely as total summer exercise increased (OR = 0.65; *P* = 0.015) (Table 5). Horses kept on non-grass turnout were more likely to be diagnosed/managed/treated for laminitis than horses kept in grass fields (OR = 2.04; *P* = 0.030). Non-significant predictors included breed, horse type, and yard type.

**Table 5.** Significant predictors for horse owner (n = 802) reported diagnosis, treatment, or management of laminitis within the preceding 12 months

Laminitis = 1; No laminitis = 0						
Independent variable	Level	Co-efficient	SE co-efficient	*P*-value	Odds ratio	95% CI
Use	Unridden	baseline				
	Ridden	–0.962	0.308	**0.002**	0.38	0.21, 0.70
Total summer exercise	Continuous	–0.426	0.175	**0.015**	0.65	0.46, 0.92
Type of summer turnout	Grass	baseline				
	Other	0.713	0.329	**0.030**	2.04	1.07, 3.89

Horses were less likely to have been diagnosed with, treated, or managed for EMS than ponies (OR = 0.31; *P* = 0.004), and ridden horses were less likely to be afflicted than unridden horses (OR = 0.27; *P* = 0.025) (Table 6). Horses kept in non-grass turnouts were more likely to be reported as having EMS than horses kept on grass (OR = 8.03; *P* < 0.001). EMS was more likely with increased winter exercise (OR = 1.24; *P* = 0.045). Non-significant predictors included breed type and total summer exercise.

**Table 6.** Significant predictors for horse owner (n = 802) reported diagnosis, treatment, or management of Equine Metabolic Syndrome within the preceding 12 months

EMS = 1; no EMS = 0						
Independent variable	Level	Co-efficient	SE co-efficient	*P*-value	Odds ratio	95% CI
Horse type	Pony	baseline				
	Horse	–1.158	0.401	**0.004**	0.314	1.43, 6.94
Use	Unridden	baseline				
	Ridden	–1.298	0.581	**0.025**	0.273	1.09, 11.01
Total winter exercise	Continuous	0.216	0.108	**0.045**	1.24	1.01, 1.53
Type of turnout (summer)	Grass	baseline				
	Other	2.083	0.444	**< 0.001**	8.03	3.36, 19.17

Natives were less likely to display locomotor stereotypies than hot-bloods (OR = 0.29; *P* < 0.001) (Table 7). Horses kept at their owner’s premises were less likely to display locomotor stereotypies than those at livery yards (OR = 0.44; *P* = 0.003). Presence of locomotor stereotypies was less likely as number of horses in summer turnouts increased (OR = 0.75; *P* = 0.022). Non-significant variables included if the horse was ridden, type of turnout, environmental complexity, and total amount of HLE.

**Table 7.** Significant predictors of horse owner (n = 802) reported locomotor stereotypies, displayed by their horse in the preceding 12 months

Locomotor stereotypy = 1; No locomotor stereotypy = 0						
Independent variable	Level	Co-efficient	SE co-efficient	*P*-value	Odds ratio	95% CI
Breed type	Hot-blood	baseline				
	Native	–1.24	0.336	**< 0.001**	0.29	0.15, 0.56
	Sports	–0.511	0.299	**0.087**	0.60	0.33, 1.08
Yard type	Livery	baseline				
	Own	–0.818	0.274	**0.003**	0.44	0.26, 0.75
	Other	–0.693	0.501	0.166	0.50	0.19, 1.33
Number of conspecifics in summer turnout	Continuous	–0.838	0.329	**0.022**	0.75	0.58, 0.96

Hot-bloods (OR = 2.50; *P* = 0.003) and sports horses (OR = 1.93; *P* = 0.009) were more likely to display aggression towards humans than natives, and unridden horses were less likely to show aggression than ridden horses (OR = 0.52; *P* = 0.044) (Table 8). Aggression became less likely as number of conspecifics in summer turnout areas increased (OR = 0.79; *P* = 0.035). Non-significant variables included total HLE and yard type.

**Table 8.** Significant predictors of horse owner (n = 802) reported aggression towards humans, displayed by their horse in the preceding 12 months

Aggression towards humans = 1; No aggression = 0						
Independent variable	Level	Co-efficient	SE co-efficient	*P*-value	Odds ratio	95% CI
Breed type	Native	baseline				
	Hot-blood	0.915	0.306	**0.003**	2.50	1.37, 4.55
	Sports	0.657	0.250	**0.009**	1.93	1.18, 3.15
Use	Ridden	Baseline				
	Unridden	–0.657	0.327	**0.044**	0.52	0.27, 0.98
Number of conspecifics in summer	Continuous	–0.237	0.113	**0.035**	0.79	0.63, 0.98

### Belief scenario

Respondents with horses at livery yards found it significantly less realistic to alter field set-ups than those whose horses were on their own properties (Χ^2^ = 105.7, df = 6; *P* < 0.001). Respondents with their own yard selected altering the field set-up as their most realistic option (n = 201), while those on livery yards either chose muzzling (n = 131) or riding more (n = 157). Similarly, those with their own properties were more likely to believe changing the field set-up would be most effective at weight management (n = 218) while those with horses on livery thought either muzzling (n = 96) or riding (n = 95) would be most effective (Χ^2^ = 21.8, df = 6; *P* = 0.001).

When respondents were asked which solution was ideal, if all were equally possible, there was no difference between solutions chosen by those on livery yards versus those with their own properties (Χ^2^ = 7.009, df = 6; *P* = 0.32). Both groups identified changing the field set-up as the most desirable option (n = 242 for livery boarders, n = 234 with own yard). There was also no difference between the two groups when asked which option they thought the horse would prefer (Χ^2^ = 10.9, df = 6; *P* = 0.09). Both groups identified changing the field set-up (n = 244 for livery boarders, n = 256 with own yard) as their top choice.

## Discussion

### Current exercise-related practices

Study results indicate that respondents’ horses are typically managed conventionally, via a combination of individual stabling and grass pasture turnout. Though all horses had access to turnout over summer months, with most living out full-time, many did receive stabling for at least a portion of each day, and over winter months, most horses were stabled regularly, with a few receiving no turnout. Respondents reported that grass fields, with relatively low environmental complexity, were the prevailing environment for their horses when not stabled. The key implications of these outcomes are two-fold. Importantly, they suggest that respondent horses are representative of the wider UK domestic horse population, as prevailing management strategies are consistent with previous findings (Hockenhull & Creighton 2014). Additionally, these results imply that a substantial number of domestic horses are kept in obesogenic environments, as grass pastures can facilitate the development of obesity (Giles *et al.* 2014) and comorbidities including laminitis (Geor 2009). As was confirmed in this study, typical turnout areas are simplistic grass fields (Rioja-Lang *et al.* 2020). Therefore, while access to these open areas facilitates SDE through free movement, the grass-based composition of these turnout areas may afford horses the opportunity to easily and substantially exceed caloric needs, as grasses are nutrient-rich and obtainable with very little energy expenditure (i.e. need or motivation to move, despite ample opportunity), particularly over the summer growing season (Longland & Byrd 2006; Wyse *et al.* 2008). This may negate some of the health benefits associated with SDE, including increased energy expenditure, due to overconsumption. Many horses received daily stabling in summer months, which is often presumed to prevent overconsumption of pasture grasses. However, previous research indicates that horses simply increase intake rates during allocated grazing times, nullifying the suggested benefits of such management (Glunk *et al.* 2013). Additionally, stabling restricts movement, further limiting opportunities for energy expenditure via SDE (Geor 2010). Overall, therefore, the typical management of respondents’ horses may contribute to excess weight gain and subsequent physical health issues, with negative consequences for welfare.

While most respondents’ horses received HLE, associated energy expenditures would be unlikely to neutralise the obesity risk promoted by grass-rich environments. Though a small proportion of horses received only unridden HLE, most were ridden. However, the most popular types of HLE (flatwork, hacking and polework) are low-intensity activities, with lesser energetic demands than activities such as jumping and interval training (Clayton 1991). This finding, particularly when combined with the modest overall levels of HLE received by most respondents’ horses, suggests that workloads are generally submaximal, limiting energy expenditures that could otherwise contribute to weight management. This inference is corroborated by Giles *et al*. (2014), who determined that low-intensity HLE was insufficient at mitigating obesity risk in their sample of UK leisure horses. Moreover, horse owners frequently overestimate their horses’ workloads (Hale *et al.* 2016). It is likely that actual exercise durations, frequencies, and intensities were lower than what was described, which is a limitation of this study. The findings related to HLE should therefore be regarded as a high estimation, further suggesting that HLE is unlikely to fully mitigate obesity risk and may consequentially be inadequate at safeguarding welfare. Collectively, findings regarding respondents’ current practices suggest that prevailing management and exercise regimes likely promote excess body condition in their horses, and that both SDE and HLE in the ways they are currently being provided are inadequate at mitigating obesity risk among horses.

Nearly 40% of respondents reported that, on average, over the past year their horse was overweight or obese. These results echo those of Wyse *et al.* (2008) who reported that 45% of surveyed riding horses in Scotland were “fat” or “very fat”. Additionally, given the propensity of owners to underestimate their horses’ body condition, and their struggles to recognise obesity (Furtado *et al.* 2020), excess weight among respondents’ horses is likely more extensive than that reported. Only 12.6% of respondents reported that their horse had been diagnosed, treated, or managed for excess weight in the past year, suggesting most horses who are owner-identified as being overweight are not being managed accordingly, which is a welfare concern. This may indicate that respondents believe excess weight to be an inherent characteristic of their horses, without recognising it as a health problem requiring treatment. This would be consistent with Furtado *et al.*’s (2020) finding that owners struggle to differentiate between obesity and the “normal shape” of their horse. Alternatively, this result may suggest that while owners realise their horses are overweight, they feel unable to intervene effectively, potentially indicating a lack of opportunity.

Regression results indicated that breed type was the only significant predictor of a horse being owner-recognised as overweight, with native types being more vulnerable than either sports horses or hot-bloods. HLE parameters (e.g. if a horse was ridden, total amount of HLE) and opportunities for SDE measured via turnout time were not correlated with being overweight. These findings are in accordance with those of Giles *et al*. (2014), who also found breed to be the strongest predictor of obesity, such that native horses/ponies were more vulnerable to obesity than other breeds. This further supports the point that the grass-based environments that facilitate SDE likely also promote overconsumption, with subsequent consequences for welfare.

While obesity is a standalone health issue, it can also contribute towards laminitis, the painful and debilitating condition involving inflammation of the hoof lamellae (Bailey & Bamford 2013). When obesity and laminitis combine with hyperinsulinaemia, the condition is collectively referred to as Equine Metabolic Syndrome (EMS) (Agne 2010). Increased exercise may contribute to prevention or management of laminitis and EMS (Geor 2010). This study found that ridden horses were less likely to have been diagnosed, treated, or managed for either laminitis or EMS than unridden horses. This may suggest that HLE aids in their prevention and also reinforces that horses afflicted with these conditions are often unable to be ridden due to lameness (Menzies-Gow *et al.* 2010). Horses with either/both of these conditions were more likely to receive alternative turnout (i.e. not grass fields) than horses without. While one might therefore presume that these alternative systems *caused* the conditions, the more likely scenario is that these systems are used to promote SDE to *manage* laminitis and EMS, in accordance with the findings of a report from the University of Liverpool, UK (Furtado *et al.* 2021). The premise of such systems is to increase SDE while facilitating species-normal socialisation and foraging behaviours (Furtado *et al.* 2021). This is particularly true of ‘Paddock Paradise’ track systems, which are low- or no-grass tracks often built around the perimeter of a field, with resources distributed throughout to encourage movement (Jackson 2006). Owners are typically prompted to implement such systems once serious health conditions, including laminitis and EMS, already affect their horses (Furtado *et al.* 2021). This likely explains why overweight horses were not more likely to receive alternative turnout than their fitter conspecifics: owners often wait until obesity triggers further complications before acknowledging the problem (Furtado *et al.* 2020).

Incidences of laminitis were negatively correlated with total summer HLE, whereas occurrences of EMS were positively correlated with total winter HLE. It is likely that increased HLE over summer helped mitigate laminitis risk in the season when it can easily occur due to high growth rates of grasses containing laminitis-provoking simple sugars (Longland & Byrd 2006). Additionally, horses suffering from laminitis would presumably not be able to be ridden. The association between EMS and HLE in winter suggests that owners of horses afflicted with EMS are motivated to provide substantial HLE during a time when respondents collectively indicated exercise levels are typically lower than in summer. Interestingly, breed type did not predict either laminitis or EMS among respondents’ horses. This suggests that all horses have the potential to develop these conditions. It is important to raise owner awareness regarding the vulnerability of all horses to these diseases, regardless of breed type, and to promote practical, accessible ways to mitigate risks, while upholding equid welfare.

Though physical health indicators were clearly correlated with exercise-related variables, the impacts of exercise on behaviour were less conclusive. Breed type was a significant predictor of both behavioural welfare indicators: presence of locomotor stereotypies, and aggression towards humans. Natives and sports horses were less likely to display locomotor stereotypies than hot-bloods, and both hot-bloods and sports horses were more likely to show aggression towards humans than natives. This is ostensibly less about differential welfare than it is about temperament as hot-bloods, whose bloodlines are the foundation of sports horses, were selectively bred for heightened reactivity, predisposing them to displays of unwanted behaviours, whereas coldblooded breeds, including natives, are comparatively stoic (McGreevy 2012).

Controlling for breed effects, horses on livery yards were more likely to display locomotor stereotypies than those kept elsewhere. Locomotor stereotypies are thought to indicate frustration at the inability to access desired resources (e.g. food) due to confinement (Hothersall & Casey 2012). Most horses kept at livery yards were on ‘DIY’ livery, meaning owners are responsible for all care, including feeding and turnout from stables to fields. Consequently, horses often have asynchronous routines and see their conspecifics fed and/or turned out without receiving these desirable resources themselves. Cooper *et al.* (2005) reported incidences of locomotor stereotypies increased when horses viewed neighbouring horses being fed but did not receive food themselves. Unpredictable and asynchronous routines that frustrate motivations to acquire resources therefore likely caused increased locomotor stereotypy presence among horses kept at livery yards.

Increased number of conspecifics in turnout areas was negatively correlated with presence of locomotor stereotypies. Horses are gregarious, so providing appropriate companions is an integral component of upholding welfare (Hartmann *et al.* 2012; Ruet *et al.* 2019). Increased access to, and choice of, conspecifics therefore likely enhanced opportunities for horses to engage in meaningful SDE through socialisation. These results also underscore agency as important contributors towards positive welfare (Littlewood *et al.* 2023). Keeping animals in appropriate groups affords them the freedom to choose with whom to interact, adding a level of social complexity and active engagement that is otherwise inaccessible (Špinka 2019). Such interactions are emphasised in the Five Domains model of welfare both as a standalone component of welfare, and as a crucial contributor to overall mental state (Mellor *et al.* 2020). Other movement-related variables, including total amount of HLE and environmental complexity, did not predict presence of locomotor stereotypies. Collectively, these results indicate that there is no substitute for socialisation.

Interestingly, ridden horses were more likely to display aggression towards humans than unridden horses. This may simply be because ridden horses are handled more often, creating greater opportunity for aggression. However, riding may involve applications of inappropriate training methods, use of ill-fitting tack, or may exacerbate any musculoskeletal or other pain-related issues (Waran *et al.* 2006; Greve & Dyson 2014, 2015; Dyson & Van Dijk 2020). Aversive training can provoke displays of conflict behaviours, including biting and kicking (McLean & Christensen 2017). A preference test conducted by König von Borstel and Keil (2012) indicated horses prefer to access feed and/or conspecifics than to be ridden. Lee *et al.* (2011) found that most horses choose to remain stabled rather than running on a treadmill but prefer being turned out into a paddock over remaining stabled, particularly if being turned out with another horse. Together, these findings suggest that HLE may not be the average horse’s preferred type of movement, which has important ramifications for welfare.

Overall, behavioural analyses stress the importance of social opportunities for welfare and highlight the effect of breed type on displays of unwanted behaviours. However, they do not provide robust evidence to either support or refute the importance of exercise on welfare. Accurate assessment of equine affective state remains a challenge among welfare scientists (Hall *et al.* 2018) and horse owners (Rioja-Lang *et al.* 2020). This study relied upon simplistic owner-reported presence of certain behaviours and consequently was unlikely to provide a robust psychological welfare assessment. Comparatively, Qualitative Behaviour Assessment (QBA) has been validated for use in horses (Hintze *et al.* 2017) and donkeys (*Equus asinus*; Minero *et al.* 2016), and should be considered in future experimental protocols to assess the emotional well-being of horses receiving variable quantities and forms of exercise.

### Owner perceptions

Lack of owner knowledge of equine needs is commonly cited as the root of mismanagement, with subsequent impacts on welfare (Horseman *et al.* 2016; Rioja-Lang *et al.* 2020). This study is among the first to take a comparatively holistic approach to understanding horse owner behaviour, using Michie *et al.*’s (2011) COM-B model of human behaviour. This model describes three main drivers of behaviour: capability, opportunity, and motivation. Capability includes psychological ability (i.e. knowledge) and physical capacity (e.g. skills) to produce a given behaviour. Opportunity refers to the extrinsic physical resources that facilitate behaviours, and social opportunities (e.g. cultural norms, peer pressures) that prompt actions or beliefs. Reflective motivation involves the plans and introspection that shape behaviour while automatic motivation describes subconscious impulses and ingrained responses. Therefore, rather than stemming solely from knowledge, human behaviours, including those relating to equine management, are more accurately the result of complex, interwoven processes that must be identified and addressed to first understand, and then change, current practice.

The study results, when framed using the COM-B model, confirm that knowledge is not the only barrier to adopting new management strategies that could potentially improve domestic horse welfare. Respondents were more likely to be satisfied with their control over turnout conditions (versus preferring more control) as turnout duration increased and as forage became increasingly available over winter, suggesting acknowledgement of species-normal needs for unstructured movement and foraging opportunity (McGreevy 2012). Respondent satisfaction was also more likely with increasing habitat complexity. This may show awareness of environmental enrichment as a means to improve welfare by facilitating movement and exploration in confinement (Mason *et al.* 2007). As the number of unwanted behaviours displayed by a horse increased, that horse’s carer became less likely to be satisfied with their control over turnout conditions, potentially demonstrating knowledge that behavioural issues communicate suboptimal welfare (Normando *et al.* 2011). Finally, respondents were more likely to be satisfied with their level of control as the number of horses sharing winter turnouts decreased. Antagonistic encounters tend to occur when forage is limited, as is likely the case in grass fields over winter, but not when sufficient forage availability prevents competition (Benhajali *et al.* 2009). Respondents seemingly acknowledge the role of limited resources in provoking competition in a species that otherwise coexists peacefully. Overall, therefore, responses were broadly in line with those that would be expected from knowledgeable horse people, potentially reflecting the high levels of experience across participants, and suggesting that a lack of knowledge is unlikely to be the sole driver of management decisions that can lead to welfare issues in horses.

Results indicate that keeping horses at a livery yard is a substantial barrier to promoting SDE, due to inopportunity. When given a choice of three equine weight-loss strategies (increasing HLE, restricting SDE through stabling and muzzling during turnout, or altering field set-ups to promote SDE), respondents with horses at livery and those with horses on their own premises both believed altered field set-ups that promote SDE were the most welfare-friendly option, as well as their ideal solution. However, those on livery felt this was less realistic than either muzzling/stabling or increasing HLE, whereas those with their own properties thought altering the field set-up was the most viable option. This variance is likely due to a lack of social and physical opportunity at livery yards, whereby control over land management, rules and sociocultural norms act as barriers (Furtado *et al.* 2020). This is an important result as it highlights the role of livery yards, and yard owners in cultivating opportunity for owners to manage their horses in ways that could be beneficial for welfare.

Interestingly, when asked which solution would be the most *effective*, chosen strategies mirrored those selected as most *realistic* within each group of respondents. This suggests horse owners may undervalue practices that they perceive to be unobtainable. This may impact uptake of welfare-enhancing recommendations throughout multiple facets of the equine industry and should therefore be the subject of future research. At present, it is unknown which of the presented weight-control strategies is actually the most effective. Muzzling has previously been shown to substantially reduce forage intake rates in ponies (Longland *et al.* 2011), though muzzles may need to be worn 24 h per day to avoid compensatory eating (Davis *et al.* 2020). It is also uncertain if horses display the same rebound effect as their smaller conspecifics. The benefits of HLE for physical health are discussed above, as is the evidence that suggests respondents are likely correct in believing increased HLE is most probably not the solution most horses would choose. Alternative management, particularly track systems, are believed to facilitate weight loss (Furtado *et al.* 2021) and their theoretical foundation is rooted in upholding welfare through facilitation of movement and species-normal behaviours (Jackson 2006). However, further research is necessary to determine if track systems increase energy expenditure in practice. Hampson *et al*.’s (2010) preliminary findings were that racetrack-style turnout did not increase movement. They also reported that complicated spiral enclosures decreased activity compared to typical rectangular paddocks. This may be because horses are adapted to open grasslands (MacFadden 1992), and therefore potentially lack cognitive adaptations to navigate complex maze-like environments. Practical applications of existing track systems are diverse in terms of land use, surfaces, enrichment, and equid density (Furtado *et al.* 2021). They therefore likely vary in success at increasing movement and exploratory behaviours. In comparison with strip-grazing, however, track systems have been shown to promote increased SDE and decreased agonistic behaviours among ponies, thus being more conducive to good welfare (Kirton *et al.* 2024). This is an encouraging finding, though further research is needed to better understand if, and how, track systems should be implemented to promote physical and emotional equine well-being.

Though livery yards appear to be a barrier to providing increased SDE, respondents with horses on livery were more likely to be satisfied with the amount of HLE their horse received than those with horses kept elsewhere. Still, 90% of respondents indicated that barriers limit their provision of HLE. Time, weather, and riding facilities were the primary barriers, suggesting an overall lack of physical opportunity to provide sufficient HLE. As most respondents were leisure owners, they presumably have numerous other demands on their time, including families and careers, which are unlikely to be modifiable in order to increase their horse’s HLE. When identifying the main reason for relocating their horse, “improved living conditions” was chosen over four times more than “improved riding facilities”, presumably reflecting respondent priorities. Collectively, these findings indicate that, although increased HLE is suggested to improve horse health (e.g. Geor 2010), this may not be a viable solution for many horse owners due to barriers that are beyond their control. Therefore, finding practical, effective ways to increase SDE is potentially a more useful path to pursue in both research and practical applications.

### Animal welfare implications

Collectively, analyses on welfare indicators suggested that increased exercise, particularly via SDE rather than HLE, is worthy of further investigation as a means to improve welfare. Our results provided preliminary indications that increased exercise can lesson risk of health conditions and show the importance of management systems that give horses opportunities to display species-normal behaviours, including socialisation. When the results of this study were contextualised within the COM-B model of human behaviour, horse owners appeared to be most limited in their provision of increased movement by opportunity, rather than knowledge or motivation. It is therefore important to acknowledge that upholding animal welfare is not only the responsibility of the horse’s direct carer, but also other potential change agents, such as livery yard owners, who are responsible for land-management decisions. Future studies directed at livery yard owners, in particular, would be useful to identify their own determinants of behaviour, to build a robust picture of the management factors contributing to horse welfare. Our results justify further research to explore ways of increasing SDE while facilitating species-normal behaviours such as foraging and socialising opportunities, paying particular attention to track systems as a potentially viable alternative management strategy.

## Conclusion

This study found that horses are typically managed in obesogenic conditions that may have welfare consequences. Evidence suggests that increased exercise correlates with improved physical health, and that facilitating species-normal behaviours including socialisation is an important aspect of psychological well-being. Owners are largely motivated to provide their horses with exercise, exploration, and freedom to express normal behaviours, and are broadly aware of associated benefits, but are frequently limited in their opportunities to do so.

## Supporting information

Naydani and Coombs supplementary material Naydani and Coombs supplementary material

## Supplementary material

The supplementary material for this article can be found at http://doi.org/10.1017/awf.2025.11.
