# Supplementary material for: Exercise as a welfare strategy? Insights from horse (Equus caballus) owners in the UK
Source: Anim Welf. 2025 Mar 4;34:e14. doi: 10.1017/awf.2025.11 (PMC11894402; doi:10.1017/awf.2025.11)
Supplement: Naydani and Coombs supplementary material [file S0962728625000119sup001.pdf]

**Supplementary Materials 1: Questionnaire**

Exercise as a welfare strategy? Insights from horse (Equus caballus) owners in the UK: Cynthia Naydani and Tamsin Coombs

# Movement as medicine: Horse owner perceptions of environment and exercise as opportunities to improve wellbeing

## Page 1: Consent Statement

**Title of Project:** Movement as medicine: Horse owner perceptions of environment and exercise as opportunities to improve welfare.

**Description of project:** This research is being carried out as a dissertation project by [redacted] for an MSc in Applied Animal Behaviour and Animal Welfare at the University of Edinburgh, supervised by [redacted]

The aim of this project is to understand how horse owners/carers in the UK manage their horses, as related to movement and exercise (e.g., turnout and ridden/ other activity). This research has received full ethical approval from the University of Edinburgh.

This questionnaire is expected to take approximately 15 minutes to answer. You will be asked basic questions about yourself and your horse/pony. You will also be asked to answer questions about how you keep your horse/pony (e.g., turnout, stabling, riding), and questions about your horse/pony's health. You will then be asked about your priorities in management factors and will be given some scenarios to think about. No personal data will be collected, and all responses are fully anonymous.

**Eligibility:**

To take part in this survey you must:

1 / 38

- Be at least 18 years of age
- Reside in the UK
- Own, share, or loan a horse or pony, and have had that horse/pony for at least one year (12 months). If you own more than one horse/pony, you will be asked to pick the horse/pony whose name comes first alphabetically and answer the questions for this animal. Please do not complete the survey more than once.

Your participation is voluntary. You may choose not to participate, and you may withdraw at any time until you click "Submit". If you decide not to participate or to withdraw from the study, you will not be penalised in any way, and your responses will not be used. Once you click "Submit" you will no longer be able to withdraw your participation due to the anonymous nature of the study.

Contact information: [redacted]

If you would like to find out the results of the study, please contact [redacted] after August 15, 2021.

By clicking the 'Yes' button, you are confirming that:

- You meet the above eligibility criteria
- You understand the purpose of this study
- You understand that you are free to withdraw your consent for involvement, however, as the survey is anonymous, once the 'submit' button is clicked, it will not be possible to withdraw
- You understand that your name will not appear in any published document relating to this study, as all information collected is completely anonymous.
- You understand that the data collected will - though fully anonymised - appear in publications, presentations, and reports relevant to this area of research.
- You understand that the data collected may be used as part of future ethically approved research.

By continuing and submitting a response to the survey, you have read and understood the above and have given informed consent.

1. Do you agree? * *Required*

- ○ Yes
- ○ No

# Page 2: Section 1: About You

Please tell us about yourself.

2. What country do you live in? ✱ *Required*

- England
- Scotland
- Wales
- Northern Ireland

3. How do you describe your gender? *Optional*

- Female
- Male
- In another way
- Prefer not to say

4. What is your age range? *Optional*

- 18-34
- 35-59
- 60+

5. What is the highest level of education you have completed, or are currently enrolled in? *Optional*

- No formal education
- GCSEs/National 5s or equivalent
- A levels/Highers or equivalent
- Further education/College/HNC/HND
- Undergraduate degree
- Postgraduate degree

6. Which of the following describe your role(s) in the equine industry? You may select more than one answer. ✱ *Required*

- Leisure owner/carer/rider (can include fun rides, riding clubs, clinics, unaffiliated competitions)
- Competitive rider (affiliated competitions)
- Professional rider/trainer/coach/instructor
- Livery yard owner/manager
- Other equine professional (e.g., stablehand, groom, farrier, saddler, physio, bodyworker, etc..)
- Equine Behaviourist
- Academic/ researcher
- Other

6.a. If you selected Other, please specify:

7. How long have you been involved with horses, excluding any long breaks of a year or more? ✱ *Required*

- 1-2 years

- 3-5 years
- 6-10 years
- 11 years or more

8. In total, how many horses/ponies have you owned, loaned, or shared over your lifetime? * *Required*

- 1
- 2-5
- 6-10
- 11 or more

9. How many horses/ponies do you currently own, share, or loan? * *Required*

- 1
- 2-5
- 6-10
- 11 or more

## Page 3: Section 2: About Your Horse

Please answer the following questions about your horse. **If you have more than one horse, for the rest of the survey, please tell us about the one whose stable name comes first alphabetically, out of the horses you have owned, loaned, or shared for at least one year.**

If you share a horse, please ensure the survey is only completed once per horse (i.e., only one sharer/owner completes the survey).

Please note that throughout the survey, the word "horse" will be used to refer to either a horse or pony.

*10.* Is your horse a: * *Required*

- Horse (over 14.2hh)
- Pony (up to and including 14.2hh)

*11.* What breed type best describes your horse? If your horse is a mix of two types please choose the one you feel best represents your horse. * *Required*

- Hot blood (Thoroughbred, Arab, Anglo-Arab)
- Sports horse/pony (e.g., warmblood, Irish Sports Horse, Thoroughbred cross, warmblood cross)
- Gaited horse
- Draft (e.g., Shire, Clydesdale)
- Native horse/pony (e.g., Cob, Welsh, Highland, Exmoor)
- Shetland pony

*12.* What sex is your horse? * *Required*

- Mare/Filly
- Gelding
- Stallion/Colt

13. How old is your horse? * *Required*

- 0-4 years
- 5-14 years
- 15-24 years
- 25 years +

14. What is your horse's main use? * *Required*

- Leisure horse - ridden (incl. riding clubs, fun rides, clinics, lessons, unaffiliated competitions)
- Leisure horse - unridden (incl. in-hand, liberty, groundwork, or no work/ companion)
- Competition horse (any discipline)
- Hunt horse
- Racehorse
- Breeding stock (broodmare or stallion)
- Young horse (not yet started work, but will be trained in the future)
- Retired horse (used to work)

15. Is your horse owned, loaned, or shared? * *Required*

- Owned

- Loaned
- Shared

## Page 4: Section 2: About Your Horse - Continued

16. Which of the following options best describes your horse? * *Required*

- a "Good do-er"/ "Easy-keeper" (i.e., easy to keep weight on/ gains weight easily)
- a "Hard-keeper" (i.e., difficult to get weight on/ loses weight easily)
- Neither (maintains condition/ neither gains nor loses weight easily)
- Unsure

17. How would you describe your horse's average body condition over the past twelve months? * *Required*

- Very underweight
- Slightly underweight
- Ideal weight
- Slightly overweight
- Very overweight/ Obese

18. Over the past 12 months, has your horse been diagnosed, treated, or managed with/for any of the following health conditions? Please also indicate whether or not a veterinarian has been involved in the diagnosis, treatment, or management of each condition at any point in time. For example, if your horse was diagnosed with laminitis two years ago, by a vet, and you still manage your horse to prevent reoccurence, please select "Yes, over 12 months ago".

| | Has your horse been diagnosed, treated, or managed for this condition within the past 12 months? * *Required* | If yes, has a veterinarian been involved with the diagnosis, treatment, or management of this condition? |
|---|---|---|

| | Yes | No | Yes, within the past 12 months | Yes, over 12 months ago | No | Unsure |
|---|---|---|---|---|---|---|
| Joint disease (e.g., arthritis) | ○ | ○ | ○ | ○ | ○ | ○ |
| Colic | ○ | ○ | ○ | ○ | ○ | ○ |
| Ulcers | ○ | ○ | ○ | ○ | ○ | ○ |
| Respiratory issues (e.g., COPD) | ○ | ○ | ○ | ○ | ○ | ○ |
| Excess weight/ obesity | ○ | ○ | ○ | ○ | ○ | ○ |
| Lack of weight/ condition | ○ | ○ | ○ | ○ | ○ | ○ |
| Laminitis | ○ | ○ | ○ | ○ | ○ | ○ |
| Equine Metabolic Syndrome (EMS) | ○ | ○ | ○ | ○ | ○ | ○ |
| PPID (Cushings) | ○ | ○ | ○ | ○ | ○ | ○ |
| Soft tissue injury (e.g., bowed tendon, suspensory injury) | ○ | ○ | ○ | ○ | ○ | ○ |
| Back problems (e.g., kissing spines) | ○ | ○ | ○ | ○ | ○ | ○ |
| Lameness - unknown cause | ○ | ○ | ○ | ○ | ○ | ○ |
| Hoof abscesses, thrush, white line disease or other hoof condition other than laminitis | ○ | ○ | ○ | ○ | ○ | ○ |

| | | | | | | |
|---|---|---|---|---|---|---|
| Skin conditions (e.g., sweet itch, mud fever) | ○ | ○ | ○ | ○ | ○ | ○ |
| Other | ○ | ○ | ○ | ○ | ○ | ○ |

19. Over the past 12 months, has your horse ever exhibited any of the following behaviours? Please select all that apply. * *Required*

- ☐ Being difficult to catch in stable or turnout
- ☐ Box walking (walking in repetitive circles) in a stable
- ☐ Fence walking in turnout
- ☐ Weaving (swaying/ rocking side to side) in the stable or turnout
- ☐ Whinnying when other horses are taken away
- ☐ Napping when being taken away from other horses in-hand or under saddle
- ☐ Self-mutilation
- ☐ Crib-biting
- ☐ Bed eating
- ☐ Wind-sucking
- ☐ Wood chewing
- ☐ Teeth grinding or tongue-chewing/biting
- ☐ Mild aggression towards humans (e.g., pinning ears, threatening to bite)
- ☐ Aggression towards other horses (e.g., biting, kicking)
- ☐ None of the above

## Page 5: Section 3: Your Horse's Living Situation

Please tell us about your horse's living situation.

20. Where do you keep your horse? * *Required*

- Livery yard
- Own property (including a rented facility or field))
- Friend or family's place
- Other

20.a. What type of livery is your horse on?

- Full livery (horse is fed, turned out, mucked out etc. by yard staff)
- Part livery (some chores are habitually done by staff, but not all)
- DIY livery (yard staff do not habitually do any chores such as mucking out, feeding, turning out/ bringing in, etc..)

21. Have you moved your horse in the last 12 months? * *Required*

- Yes
- No

22. The last time you moved your horse (even if it was more than a year ago), what was the main reason for moving him/her? * *Required*

- Location (e.g., you moved home, or wanted your horse closer to you)
- To get better riding facilities (e.g., arena, hacking access)

- To get better living conditions for your horse (e.g., turnout, stabling, other horses)
- To get better facilities (e.g., tack storage, trailer parking, solarium)
- To get better yard management/ horse handling by staff
- To change livery type (e.g., full livery to DIY, or vice versa)
- Other
- N/A - I have never moved my horse

22.a. If you selected Other, please specify:

## Page 6: Section 3: Your Horse's Living Situation - Continued

Please tell us about your horse's turnout. For the following questions, "Turnout" refers to any area where your horse is not individually housed in a stable. For example, if your horse has free access to a field shelter or communal barn, this *would* count as part of turnout time/area.

*23.* In the summer, what type of turnout does your horse typically have access to? * *Required*

- Grass area (field/pasture/paddock)
- Track
- Equi-central system
- Woodlands/Moorland
- Dry lot/hard standing paddock
- No turnout

*23.a.* How many hours each day does your horse typically have access to turnout in summer?

- Up to 4 hours a day
- 4-8 hours a day
- 8-16 hours a day
- 16-24 hours a day
- 24 hours/day

*23.b.* How satisfied are you with the duration of time your horse spends turned out in summer? * *Required*

- I am satisfied
- I would prefer more turnout time

- I would prefer less turnout time

23.c. What is the size of this turnout area?

- Less than 1 acre
- 1-3 acres
- 3-10 acres
- Over 10 acres
- Unsure

23.d. How satisfied are you with the amount of space your horse has in their summer turnout?

- I am satisfied
- I would prefer more space
- I would prefer less space

23.e. How many other horses does your horse usually get turned out with in summer?

- No other horses
- 1 other horse
- 2-4 other horses
- 5 or more other horses

23.f. How satisfied are you with the number of other horses your horse is turned out with in summer?

- I am satisfied
- I would prefer more other horses
- I would prefer less other horses

23.g. Which of the following environmental features, if any, are present in your horse's summer turnout space? Please select all that apply.

- ☐ Man-made field shelter
- ☐ Trees
- ☐ Bushes, shrubs, and/or hedgerows
- ☐ Forage slow feeders (e.g., hay nets)
- ☐ Scratching posts
- ☐ Ponds and/or streams
- ☐ Co-grazers such as sheep or goats
- ☐ Toys
- ☐ Logs, poles, or other obstacles
- ☐ Sandpits
- ☐ None of the above

23.h. How satisfied are you with the amount and type of environmental features available in your horse's summer turnout?

- ○ I am satisfied
- ○ I would prefer more environmental features
- ○ I would prefer less environmental features

23.i. Please rate your agreement with the following statements about your horse's summer turnout.

Please don't select more than 1 answer(s) per row.

Please select at least 1 answer(s).

| | Strongly disagree | Disagree | Neither agree nor disagree | Agree | Strongly agree | Unsure/ Not applicable |
|---|---|---|---|---|---|---|

| | | | | | | |
|---|---|---|---|---|---|---|
| My horse uses most of the environmental features in the turnout area often | ☐ | ☐ | ☐ | ☐ | ☐ | ☐ |
| The environmental features are important for my horse's physical wellbeing | ☐ | ☐ | ☐ | ☐ | ☐ | ☐ |
| The environmental features are important for my horse's emotional wellbeing | ☐ | ☐ | ☐ | ☐ | ☐ | ☐ |
| The environmental features encourage my horse to increase movement | ☐ | ☐ | ☐ | ☐ | ☐ | ☐ |
| The design of the turnout area itself encourages my horse to increase movement | ☐ | ☐ | ☐ | ☐ | ☐ | ☐ |
| My horse often waits at the gate to come in | ☐ | ☐ | ☐ | ☐ | ☐ | ☐ |

| | | | | | | |
|---|---|---|---|---|---|---|
| My horse often trots, canters, or gallops out of playfulness while turned out | ◻ | ◻ | ◻ | ◻ | ◻ | ◻ |
| My horse often trots, canters, or gallops due to stress while turned out | ◻ | ◻ | ◻ | ◻ | ◻ | ◻ |
| My horse grooms with other horses while turned out | ◻ | ◻ | ◻ | ◻ | ◻ | ◻ |
| My horse plays with other horses while turned out | ◻ | ◻ | ◻ | ◻ | ◻ | ◻ |

24. In the winter, what type of turnout does your horse typically have access to? *
*Required*

- ◯ Grass area (field/pasture/paddock)
- ◯ Track
- ◯ Equi-central system
- ◯ Woodlands/Moorland
- ◯ Dry lot/hard standing paddock
- ◯ No turnout

24.a. How many hours each day does your horse typically have access to turnout in winter?

- ◯ Up to 4 hours a day

- 4-8 hours a day
- 8-16 hours a day
- 16-24 hours a day
- 24 hours/day

24.b. How satisfied are you with the duration of time your horse spends turned out in winter? * *Required*

- I am satisfied
- I would prefer more turnout time
- I would prefer less turnout time

24.c. What is the size of this turnout area?

- Less than 1 acre
- 1-3 acres
- 3-10 acres
- Over 10 acres
- Unsure

24.d. How satisfied are you with the amount of space your horse has in their winter turnout?

- I am satisfied
- I would prefer more space
- I would prefer less space

24.e. How many other horses does your horse usually get turned out with in winter?

- No other horses
- 1 other horse
- 2-4 other horses

- 5 or more other horses

24.f. How satisfied are you with the number of other horses your horse is turned out with in winter?

- I am satisfied
- I would prefer more other horses
- I would prefer less other horses

24.g. Which of the following environmental features, if any, are present in your horse's winter turnout space? Please select all that apply.

- Man-made field shelter
- Trees
- Bushes, shrubs, and/or hedgerows
- Forage slow feeders (e.g., hay nets)
- Scratching posts
- Ponds and/or streams
- Co-grazers such as sheep or goats
- Toys
- Logs, poles, or other obstacles
- Sandpits
- None of the above

24.h. How satisfied are you with the amount and type of environmental features available in your horse's winter turnout?

- I am satisfied
- I would prefer more environmental features
- I would prefer less environmental features

24.i. Please rate your agreement with the following statements about your horse's

winter turnout.

Please don't select more than 1 answer(s) per row.

Please select at least 1 answer(s).

| | Strongly disagree | Disagree | Neither agree nor disagree | Agree | Strongly agree | Unsure/ Not applicable |
|---|---|---|---|---|---|---|
| My horse uses most of the environmental features in the turnout area often | ☐ | ☐ | ☐ | ☐ | ☐ | ☐ |
| The environmental features are important for my horse's physical wellbeing | ☐ | ☐ | ☐ | ☐ | ☐ | ☐ |
| The environmental features are important for my horse's emotional wellbeing | ☐ | ☐ | ☐ | ☐ | ☐ | ☐ |
| The environmental features encourage my horse to increase movement | ☐ | ☐ | ☐ | ☐ | ☐ | ☐ |

| | | | | | | |
|---|---|---|---|---|---|---|
| The design of the turnout area itself encourages my horse to increase movement | ☐ | ☐ | ☐ | ☐ | ☐ | ☐ |
| My horse often waits at the gate to come in | ☐ | ☐ | ☐ | ☐ | ☐ | ☐ |
| My horse often trots, canters, or gallops out of playfulness while turned out | ☐ | ☐ | ☐ | ☐ | ☐ | ☐ |
| My horse often trots, canters, or gallops due to stress while turned out | ☐ | ☐ | ☐ | ☐ | ☐ | ☐ |
| My horse grooms other horses while turned out | ☐ | ☐ | ☐ | ☐ | ☐ | ☐ |
| My horse plays with other horses while turned out | ☐ | ☐ | ☐ | ☐ | ☐ | ☐ |

25. Who makes decisions for your horse regarding type of turnout, duration of turnout, and who your horse is turned out with? Please select the best response. * *Required*

- ○ I make all decisions for my horse myself
- ○ I have a lot of decision making power, but someone else makes some of the decisions

- I have some decision making power, but someone else makes most of the decisions
- Someone else makes all the decisions

25.a. How satisfied are you with the amount of control you have over your horse's turnout conditions? * *Required*

- I am satisfied
- I would prefer more control
- I would prefer less control

## Page 7: Section 4: Feeding Your Horse

Please tell us about your horse's diet.

26. In the summer, what best describes the forage component of your horse's diet? * *Required*

- Grass only
- Grass and hay/haylage/other preserved forage (e.g., hay cubes, pellets, chaff)
- Hay/haylage only

26.a. How satisfied are you with the type of forage your horse gets in summer? * *Required*

- I am satisfied
- I would prefer more grass and less hay/preserved forage
- I would prefer less grass and more preserved hay/forage

26.b. How often does your horse have access to forage in the summer? * *Required*

- All of the time (ad-lib)
- A lot of the time (available more than 16 hours per day)
- Some of the time (available 8-16 hours per day)
- Not often (available less than 8 hours per day)

26.c. How satisfied are you with the availability of forage in the summer? * *Required*

- I am satisfied
- I would like my horse to have access to forage for a greater amount of time each day
- I would like my horse to have access to forage for a lesser amount of time each day

27. In the winter, what best describes the forage component of your horse's diet? * *Required*

- Grass only
- Grass and hay/haylage/other preserved forage (e.g., hay cubes, pellets, chaff)
- Hay/haylage only

27.a. How satisfied are you with the type of forage your horse gets in winter? * *Required*

- I am satisfied
- I would prefer more grass and less hay/preserved forage
- I would prefer less grass and more preserved hay/forage

27.b. How often does your horse have access to forage in the winter? * *Required*

- All of the time (ad-lib)
- A lot of the time (available more than 16 hours per day)
- Some of the time (available 8-16 hours per day)
- Not often (available less than 8 hours per day)

27.c. How satisfied are you with the availability of forage in the winter? * *Required*

- I am satisfied
- I would like my horse to have access to forage for a greater amount of time each day
- I would like my horse to have access to forage for a lesser amount of time each day

28. Who makes feeding decisions for your horse including type of forage, availability of forage, and additional feeds? Please select the best response. * Required

- ○ I make all decisions for my horse myself
- ○ I have a lot of decision making power, but someone else makes some of the decisions
- ○ Someone else makes most of the decisions but I have some say
- ○ Someone else makes all the decisions

28.a. How satisfied are you with the amount of control you have over feeding your horse? * Required

- ○ I am satisfied
- ○ I would prefer more control
- ○ I would prefer less control

## Page 8: Section 5: Exercising Your Horse

Please tell us about exercising your horse.

29. Which types of exercise does your horse take part in over summer? * *Required*

- ☐ Flatwork/ dressage
- ☐ Showjumping/ arena jumping
- ☐ Cross country jumping
- ☐ Polework
- ☐ Conditioning work (e.g., gallop sets, interval training, hillwork)
- ☐ Hunting or team chasing
- ☐ Showing: ridden
- ☐ Showing: in-hand
- ☐ Driving
- ☐ Hacking
- ☐ Endurance riding
- ☐ Multi--day trekking
- ☐ Lunging/ long-lining
- ☐ Other in-hand exercise (e.g., liberty, groundwork, leading on walks)
- ☐ Other exercise
- ☐ None of the above - horse is not in any work

29.a. In the summer, how often do you exercise your horse?

- ○ Less than once a month
- ○ Once a month
- ○ Once a fortnight
- ○ 1-2 days per week
- ○ 3-5 days per week
- ○ 6-7 days per week

29.b. How long, on average, does a summer exercise session last?

- Under half an hour
- 30-60 minutes
- Over an hour

29.c. What is the average distance covered during a summer exercise session? If you use an app such as Equilab to track rides, or other distance measuring tool, please select the best response. Otherwise, please select "unsure".

- Less than 2km
- 2-5km
- 5-10km
- Over 10km
- Unsure

29.d. On average, how intense are the summer exercise sessions?

- Low intensity - mainly walk, some trot
- Moderate intensity - mainly trot, some walk and canter
- High intensity - lots of canter, some jumping
- Very high intensity - mainly fast canter, strenuous jumping and/or galloping

29.e. How satisfied are you with the amount of exercise your horse receives in summer? ✱ *Required*

- I am satisfied
- I would like my horse to get more exercise
- I would like my horse to get less exercise

30. Which types of exercise does your horse take part in over winter? * *Required*

- ☐ Flatwork/ dressage
- ☐ Showjumping/ arena jumping
- ☐ Cross country jumping
- ☐ Polework
- ☐ Conditioning work (e.g., gallop sets, interval training, hillwork)
- ☐ Hunting or team chasing
- ☐ Showing: ridden
- ☐ Showing: in-hand
- ☐ Driving
- ☐ Hacking
- ☐ Endurance riding
- ☐ Multi--day trekking
- ☐ Lunging/ long-lining
- ☐ Other in-hand exercise (e.g., liberty, groundwork, leading on walks)
- ☐ Other exercise
- ☐ None of the above - horse is not in any work

30.a. In the winter, how often do you exercise your horse?

- ○ Less than once a month
- ○ Once a month
- ○ Once a fortnight
- ○ 1-2 days per week
- ○ 3-5 days per week
- ○ 6-7 days per week

30.b. How long, on average, does a winter exercise session last?

- ○ Under half an hour
- ○ 30-60 minutes

- Over an hour

30.c. What is the average distance covered during a winter exercise session? If you use an app such as Equilab to track rides, or other distance measuring tool, please select the best response. Otherwise, please select "unsure".

- Less than 2km
- 2-5km
- 5-10km
- Over 10km
- Unsure

30.d. On average, how intense are the winter exercise sessions?

- Low intensity - mainly walk, some trot
- Moderate intensity - mainly trot, some walk and canter
- High intensity - lots of canter, some jumping
- Very high intensity - mainly fast canter, strenuous jumping and/or galloping

30.e. How satisfied are you with the amount of exercise your horse receives in winter? * *Required*

- I am satisfied
- I would like my horse to get more exercise
- I would like my horse to get less exercise
- Unsure

31. Which of the following factors substantially limit how much you exercise your horse throughout the year? Please select all that apply. * *Required*

- [ ] Access to facilities/ suitable areas to exercise
- [ ] Access to other people to ride with (including an instructor/coach)
- [ ] Time
- [ ] Weather
- [ ] Horse age
- [ ] Horse health/soundness
- [ ] Horse behaviour
- [ ] Rider/handler health or fitness
- [ ] Rider/handler confidence or skill
- [ ] General desire/ motivation to ride or exercise your horse
- [ ] Other
- [ ] None of the above - I can exercise my horse as much as I want year-round

*31.a.* If you selected Other, please specify:

## Page 9: Section 6: Your Beliefs and Preferences

Please answer the following questions to tell us about your beliefs and preferences.

32. Please rank your level of agreement for the following statements. * Required

Please don't select more than 1 answer(s) per row.

Please select at least 10 answer(s).

| | Strongly disagree | Disagree | Neither agree nor disagree | Agree | Strongly agree | Unsure |
|---|---|---|---|---|---|---|
| I think my horse is happy with his/her living conditions | ☐ | ☐ | ☐ | ☐ | ☐ | ☐ |
| It is important for horses to be able to express natural behaviours | ☐ | ☐ | ☐ | ☐ | ☐ | ☐ |
| I think my horse enjoys his/her exercise routine | ☐ | ☐ | ☐ | ☐ | ☐ | ☐ |
| It is important for horses to live in herds | ☐ | ☐ | ☐ | ☐ | ☐ | ☐ |
| I think my horse enjoys being stabled | ☐ | ☐ | ☐ | ☐ | ☐ | ☐ |
| I think my horse enjoys being turned out | ☐ | ☐ | ☐ | ☐ | ☐ | ☐ |

| | | | | | | |
|---|---|---|---|---|---|---|
| Modern horses have different needs than wild/feral horses | ☐ | ☐ | ☐ | ☐ | ☐ | ☐ |
| I worry about my horse's weight | ☐ | ☐ | ☐ | ☐ | ☐ | ☐ |
| For horses in general, diet works better than exercise at managing weight | ☐ | ☐ | ☐ | ☐ | ☐ | ☐ |
| My horse prefers being turned out rather than stabled | ☐ | ☐ | ☐ | ☐ | ☐ | ☐ |
| It is important that my horse has access to forage at all times | ☐ | ☐ | ☐ | ☐ | ☐ | ☐ |
| I struggle to know what is best for my horse | ☐ | ☐ | ☐ | ☐ | ☐ | ☐ |
| For horses in general, physical health is more important than mental health | ☐ | ☐ | ☐ | ☐ | ☐ | ☐ |
| I feel limited in my ability to take good care of my horse | ☐ | ☐ | ☐ | ☐ | ☐ | ☐ |
| I want my horse to be warm and comfortable over winter | ☐ | ☐ | ☐ | ☐ | ☐ | ☐ |

| | | | | | | |
|---|---|---|---|---|---|---|
| I worry about my horse's welfare | ☐ | ☐ | ☐ | ☐ | ☐ | ☐ |
| I believe increasing my horse's activity levels would improve his/her welfare | ☐ | ☐ | ☐ | ☐ | ☐ | ☐ |

33. When you need to make a decision regarding your horse's care, who/what are your most relied upon sources of information to help you make that decision? Please select up to three options. * *Required*

Please select between 1 and 3 answers.

- ☐ Yard owner/manager
- ☐ Friends/family
- ☐ Veterinarian
- ☐ Riding instructor/coach/trainer
- ☐ Equine behaviourist
- ☐ Other equine professional (e.g., farrier, saddle fitter, physio)
- ☐ Books or magazines
- ☐ Scientific research/publications
- ☐ Social media groups/pages
- ☐ Industry websites (e.g., thehorse.com, Horse & Hound)
- ☐ Other

33.a. If you selected Other, please specify:

**Please consider the following potential real-world scenario, and then answer the questions that follow.**

*Scenario:* You have a 10 year old gelding who you currently ride around three times a week, for about half an hour each session. He lives out 24/7 in a grass field with three other horses who he gets on well with. Your vet attends to your horse to give him his yearly vaccinations and dental. Your vet mentions that while your horse is otherwise healthy, he is carrying excess weight which puts him at potential risk of laminitis. Your vet then advises you to work on reducing your horse's weight, and suggests the following three ways to do this:

**a.** Keep your horse's living conditions the same, but increase the amount of times you ride each week and ride him harder, for longer, each time.

**b.** Ride your horse the same amount, but bring him into a stable during the day to get him off the grass, giving him soaked hay instead, and then turning him out overnight with a grazing muzzle on.

**c.** Change your field set up so your horse and his herd have limited access to grass, and have to move from place to place to access different resources such as hay, water, and shelter. Your horse stays out 24/7 and your riding habits do not change.

34. Please answer the following questions for the above scenario: * *Required*

Please don't select more than 1 answer(s) per row.

Please select at least 3 answer(s).

| | a. Increase ridden exercise | b. Stable during the day + grazing muzzle overnight | c. Change the field setup | Unsure |
|---|---|---|---|---|
| Which of the above options do you think would be most effective? In other words, which do you think will do the best job at reducing your horse's weight? | ☐ | ☐ | ☐ | ☐ |

| | | | | |
|---|---|---|---|---|
| Which of the above options do you think would be most realistic for you to implement in your current situation (i.e., your horse's current home and your current life)? | ☐ | ☐ | ☐ | ☐ |
| Which of the above options do you think your horse would prefer? | ☐ | ☐ | ☐ | ☐ |
| Which of the above options would you most want to implement, in an ideal world, if each was equally possible? | ☐ | ☐ | ☐ | ☐ |

## Page 10: Final page

Thank you for taking the time to participate in this survey. Your input is appreciated!

If you have any questions please contact [redacted]

If you would like to find out the results of the study, please contact [redacted] after August 15, 2021.

38 / 38
